# Systematic revision of the Taiwanese genus *Kurixalus* members with a description of two new endemic species (Anura, Rhacophoridae)

**DOI:** 10.3897/zookeys.557.6131

**Published:** 2016-01-28

**Authors:** Shu-Ping Wu, Chuan-Chin Huang, Chi-Li Tsai, Te-En Lin, Jhih-Jia Jhang, Sheng-Hai Wu

**Affiliations:** 1 Department of Earth and Life Science, University of Taipei. No.1, Ai-Guo West Road, Taipei, 10048 Taiwan; 2 Department of Anesthesiology, Perioperative and Pain Medicine at Brigham and Women’s Hospital, Boston, 02115, USA; 3 Taiwan Endemic Species Research Institute, 1, Ming-shen East Road, Chichi Township, Nantou County 55244, Taiwan; 4 Department of Life Sciences, National Chung-Hsing University, No. 250, Guo-Guang Road, Taichung City, 40227 Taiwan

**Keywords:** Kurixalus berylliniris sp. n., Kurixalus wangi sp. n., oophagous tadpoles

## Abstract

Two new species of rhacophorid tree frog were identified in Taiwan. In both new taxa, derived reproductive characteristics of laying eggs in tree holes and oophagous tadpoles are shared with *Kurixalus eiffingeri*, but they are divergent from each other in molecular genetics, mating calls, and tadpole and adult morphology. The morphological characteristics and the molecular phylogenetic evidence support the hypothesis that the two new species, *Kurixalus berylliniris* **sp. n.** and *Kurixalus wangi* **sp. n.**, are both monophyletic lineages.

## Introduction

There are four genera (*Buergeria*, Tschudi, 1838, *Kurixalus*, Ye, Fei, and Dubois In Fei, 1999, *Polypedates*, Tschudi, 1838, and *Rhacophorus*, Kuhl and Van Hasselt, 1822) and eleven species of rhacophorid tree frogs on the island of Taiwan (Lue et al. 1999, Shang 2010). In 1999, Ye et al. described the monotypic genus of *Kurixalus*, which only contained *Kurixalus eiffingeri* (Fei 1999). Subsequently, new species attributed to the genus *Kurixalus* were identified and characterized in southern Asia and China (Wilkinson et al. 2002, Frost et al. 2006, Li et al. 2008, Li et al. 2009, Hertwig et al. 2013, Yu et al. 2013, Frost 2014, Nguyen et al. 2014a, Nguyen et al. 2014b) mainly based on molecular analyses. *Rana eiffingeri* was originally described by Boettger (1895), based on specimens collected from the “Liukiu -Inseln” (Boettger 1895). At present, this species is distributed on the two isles Iriomote and Ishigaki in the Yaeyama Archipaleago of Ryukyu Islands, Japan (Maeda and Matsui 1989) and the lowland to the medium elevations forests of Taiwan (Lue et al. 1999).

*Kurixalus eiffingeri*, a native species in the island of Taiwan, is the only rhacophorid within the genus *Kurixalus* that has a tree-hole breeding reproductive mode and oophagous tadpoles (Ueda 1986, Lehtinen and Nussbaum 2003, Wells 2007). *Kurixalus idiootocus*, a species endemic to Taiwan, has a lentic feeding tadpole type, which is similar to most species in the genus *Kurixalus* (Inger 1966, Inger et al. 1999, Kuramoto and Wang 1987). In previous molecular phylogenetic studies, *Kurixalus eiffingeri* and *Kurixalus idiootocus* have been recovered as sister taxa (Abraham et al. 2013, Yu et al 2013, Nguyen et al. 2014a, b). Since *Kurixalus eiffingeri* and *Kurixalus idiootocus* are the only two species that have been described from the island of Taiwan, rhacophorid frogs with similar life history to *Kurixalus idiootocus* (but see Abraham et al 2013), specifically any rhacophorid frogs with tree-hole breeding reproductive mode or lentic feeding tadpole type would be would be assigned to either of the two species.

In our study, *Kurixalus* treefrog specimens were collected from the island of Taiwan. Additionally, the specimens of *Kurixalus eiffingeri* were collected from the type localities, Iriomote and Ishigaki isles. In the field, we noticed that some of the *Kurixalus* populations in eastern and southern Taiwan resembled *Kurixalus eiffingeri* in external morphology but differed in their reproductive season (November to February) from *Kurixalus eiffingeri* (from February to August). One group has an extraordinarily small body size, and the other group has green irises. Further examination of the samples from the two populations and *Kurixalus eiffingeri* revealed the differences in external morphology, tadpole morphology, comparative anatomy, mating call analysis, and molecular genetic evidence. From these results, the two populations of rhacophorid frogs are describe as new species.

## Materials and methods

### Sampling

**Figure 1.**
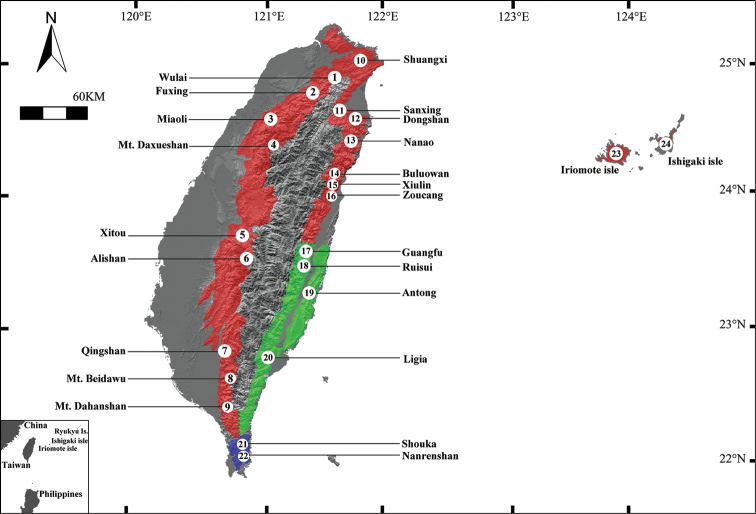
Sampling localities of this study. Localities 1 through 22 are around Taiwan island, locality 23 from Iriomote isle, locality 24 from Ishigaki isle. The two isles belong to the southern end of Ryukyu archipelago. Color refers to the geographical distribution of the three *Kurixalus* species. Red: *Kurixalus eiffingeri*; Green: *Kurixalus berylliniris* sp. n. (Taxon 1); B: *Kurixalus wangi* sp. n. (Taxon 2). Loc. 20: Ligia, type locality of *Kurixalus berylliniris* sp. n.; Loc. 21: Shouka, type locality of *Kurixalus wangi* sp. n.

The type specimens of frogs and tadpoles of the two new *Kurixalus* species were collected by hand, euthanized using a dilute chloretone solution, and fixed in 10% buffered formalin. Frogs were later transferred to 70% ethanol, and tadpoles were stored in 10% buffered formalin. In addition to the type specimens described in this study, 343 samples that consisted of *Kurixalus eiffingeri* and related taxa were collected from 22 locations throughout the island of Taiwan. Furthermore, three specimen of *Kurixalus eiffingeri* were collected from the type locality. One was from Iriomote isle and the other two were from Ishigaki isle (Fig. 1). Based on body size, mating call differences, and iris color, the samples were subdivided into three groups: *Kurixalus eiffingeri*, Taxon 1, and Taxon 2 (Fig. 1).

Morphometric characteristics of adult specimens: snout-vent length (SVL), head width (HW), head length (HL), internarial distance (IN), eye-narial distance (EN), horizontal eye diameter (ED), distance between the anterior margins of eyes (DFE), distance between the posterior margins of eyes (DBE), upper eyelid width (UEW), interorbital distance (IO), tympanic annulus diameter (TAD), distance between the axillae, between posterior margins of the upper arm (AXI), axilla-groin distance (AGD), forearm length (UAW), manus length (PAL), length of first finger from base of palmar tubercle to tip of third finger disc (F1L), width of third finger disc (D3L), femur length (FEL), tibia length (TBL), tarsus length (TSL), foot length from proximal margin of inner metatarsal tubercle to tip of fourth toe (FOL), first toe length (TL), inner metatarsal tubercle length (IML), and disc width of fourth toe (T4D) (Table S1). The abbreviations listed above were chosen to be consistent with Manamendra-Arachchi and Pethiyagoda (2005).

In addition, the eggs and tadpole morphometric characteristics were measured comprising total length (TL), body length (BL), tail length (CL), tail height (TH), tail muscle height (TM), internarial distance (NA), distance between eyes (IN), and tail muscle width (MW) (Altig and McDiarmid 1999a, b). Except for TL, BL, and CL, which were measured using dial calipers, tadpoles and eggs were measured under a dissecting microscope with a stage micrometer. Developmental stages of tadpoles were as defined by Gosner (1960). Drawings of the tadpoles were done by SHW using a dissecting microscope with a *camera lucida* attachment.

All measurements of morphometric characteristics were taken using a dial caliper under a dissecting microscope, and measurements were rounded to 0.1 mm. Digital webbing of the adults was recorded using Savage and Heyer’s formula (1997).

T-tests were used to examine whether body size varied by gender within each taxon. An analysis of covariance (ANCOVA) method was used to compare the size-adjusted means of morphometric characteristics. Morphometric characteristics that satisfied the normality assumption were included in a multivariate principal component analysis (PCA) based on the correlation matrix of size-standardized measurements (all measurements divided by SVL). Scatter plots of the scores of the first three factors of PCA were used to examine the differentiation among specimens. All of these tests and analyses were applied separately to male and female specimens. The statistical analyses were performed using SigmaPlot 12 (Systat Software, Inc.).

### Mating calls study

Frog mating calls were recorded using a digital recorder (Fostex FR-2LE) and a microphone (Sennheiser ME 67/k6). Calls were recorded in the native habitats of these tree frogs, and environmental parameters including temperature and humidity were also recorded. Avisoft SASLab Pro 5.2.08 (Avisoft Bioacoustics) was used to extract the maximum and minimum frequencies, as well as the width of frequency, the single note duration, and the time interval between notes of the mating calls. A rapid call and a slow call were identified. Slow mating calls were compared among the subtypes in a pair-wise manner using Wilcoxon-Mann-Whitney odds (WMWodds) calculations (Divine et al. 2013). A bootstrap method was used to calculate the Bonferroni corrected confidence intervals of the WMWodds.

### Molecular study

Whole genomic DNA was extracted from muscle tissue of fresh or ethanol-preserved specimens using the procedure originally described by Truett et al. (2000). We selected the mitochondrial DNA cytochrome *c* oxidase subunit 1 (CO1) and the 16S rRNA genes to examine the phylogenetic relationships among the three subtypes (S2 and Table S3). The fragments of partial CO1 (658 base pairs) and 16S rRNA (549 base pairs) genes were amplified using the primer pairs LCO1491/HCO2198 (Folmer et al. 1994) and 16Sar/16Sbr (Palumbi 1996, Vences et al. 2005, Vences et al. 2005). Each 50 μl PCR mixture consisted of 5 μl 10X reaction buffer containing 15 mM MgCl_2_, 4 μl dNTP (2.5 mM), 0.05 units of *Taq* polymerase (Super-Therm), 0.5 μl of each primer (10 pm/λ), 1 μl template DNA and ddH_2_O. Thermal cycling was performed on a GeneAmp 9700 with 5 minutes at 95 °C for pre-denaturing, 35 cycles of 1 minute at 95 °C, 1 minute at 50 °C, 1 minute at 72 °C, and a final extension for 7 minutes at 72°C for both of the CO1 and the 16S rRNA genes. The amplicons were examined on a 2% agarose gel for quality and fragment size. Then they were purified using a Geneaid PCR Extraction Kit and sequenced on an ABI 3730 automated sequencer. Estimates of genetic divergence among taxa were calculated using the Kimura two-parameter model of correction for multiple substitutions at a site (Kimura 1980). The transition / transversion ratio was set as 2:1. Chromatographs and sequences were examined and edited in BIOEDIT 7.0.1. (Hall 1999) and were aligned using CLUSTAL W (Thompson et al. 1994). The homogeneity of the two datasets was analyzed by the ILD procedure (Farris 1995) as implemented in PAUP (Swofford 2002) using the branch-and-bound search algorithm with 1000 permutation replicates to generate the null distribution. The fraction of ILD null replicates with a significance value greater than the significance value of the ILD was recorded. The sequences of the two gene segments were combined into one data set for subsequent analyses. DnaSP 5.10 (Rozas and Rozas 1999) was used to compute the population divergence conditions. TCS 1.21 (Clement et al. 2000) was used to reconstruct the minimum spanning network of haplotypes from each genetic population or species. A consensus ML tree was reconstructed by Mega 6 (Kumar et al. 2008, Tamura et al. 2013) to understand the genetic variation among the haplotypes. Three taxa, *Kurixalus idiootocus*, *Feihyla palpebralis*, and *Rhacophorus moltrechti* were used as outgroups in the phylogenetic analysis.

A general time reversible model with a proportion of invariable sites and a gamma shaped distribution of rates across sites (GTR + I + G, I = 0.4402, G = 0.4519) was determined as the best-fitting model for the aligned sequences of the combined dataset using a hierarchical likelihood ratio test performed with the program MrModeltest 2.2 (Nylander 2004). The selected substitution model then was adopted in the reconstruction of the phylogeny by Bayesian analysis and neighbor-joining (NJ) analysis (Saito and Nei 1987).

The Bayesian tree and the posterior probability distribution were determined using the program MrBayes 3.1 (Huelsenbeck and Ronquist 2001, Ronquist and Huelsenbeck 2003). Two independent Monte Carlo Markov Chain (MCMC) analyses were run simultaneously for 200,000 generations and sampled every 100 generations. To summarize the parameters and trees, the first 500 (25%) parameter values and trees were discarded. The NJ analysis was conducted in PAUP using ML distance. Gaps within the alignment were considered as missing values. The MP (Maximum Parsimony) analysis was performed with a heuristic search using 10 random stepwise steps followed by tree bisection reconnection (TBR) branch swapping. Support for nodes was evaluated by bootstrap analysis (Felsenstein 1985) with 1000 replicates of the NJ and the MP methods.

Partial sequences of mtDNA CO1 gene were used as haplotypes to examine the genetic structures of the three subtypes. We calculated the *Fst*, Nm (number of immigrants per generation, *Nm* =((1/*Fst*)-1)/4), nucleotide diversity (Pi), and haplotype diversity (Hd). These calculations were made to comprehend the divergence and the intensity of gene flow among these taxa and to infer the evolutionary histories experienced by these taxa or their populations (Wright 1965, Avise 1994).

## Results

### Systematics

#### Kurixalus berylliniris sp. n.

Taxon classificationAnimaliaAnuraRhacophoridae

http://zoobank.org/837AE8BF-FF6F-4E27-8849-20EF9E89CA0C

**Figure 2.**
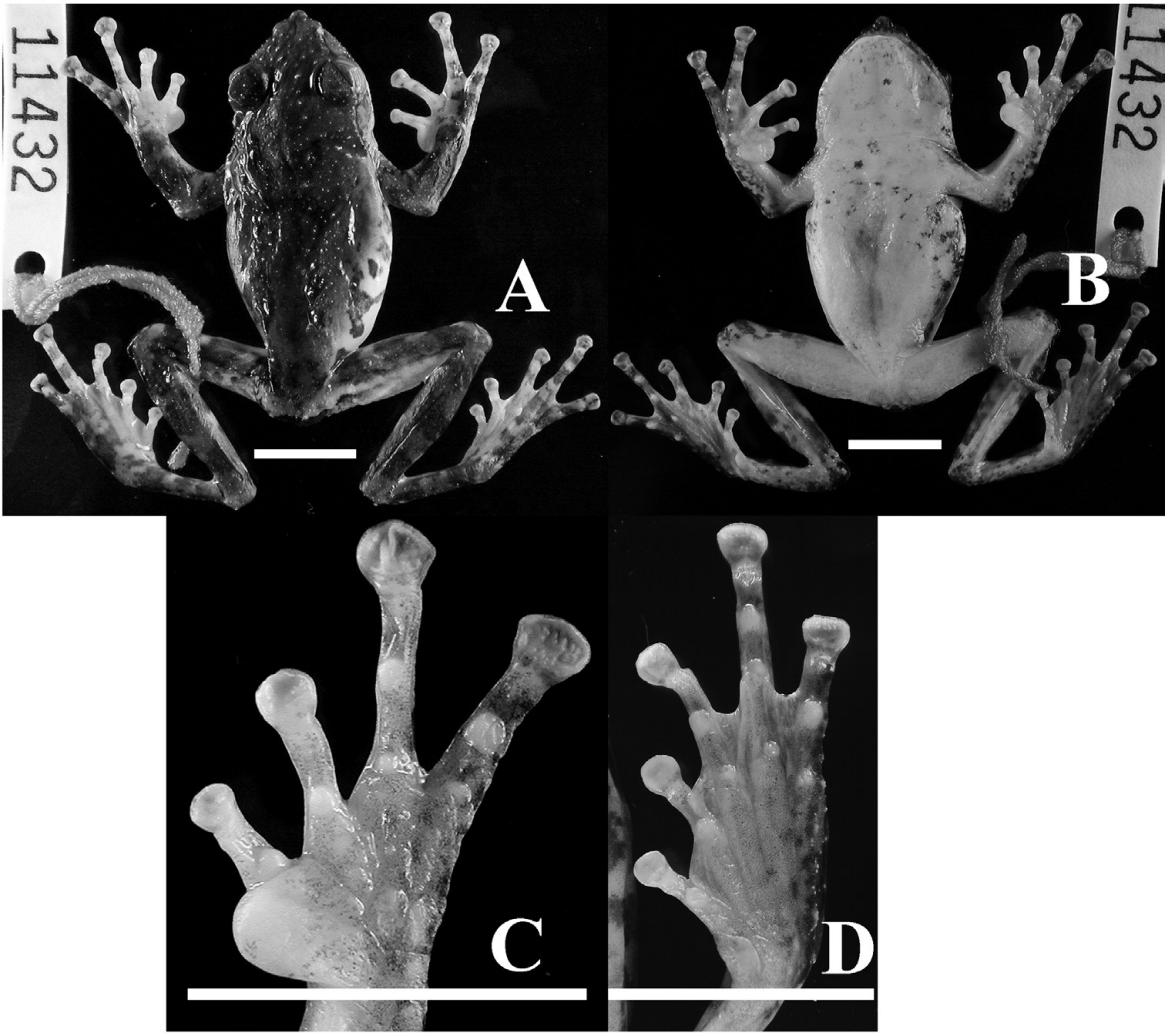
Holotype of *Kurixalus berylliniris* sp. n. Dorsal (**A**), ventral (**B**), and ventral view of hand (**C**) and foot (**D**). Scale bars: 10 mm.

**Figure 3.**
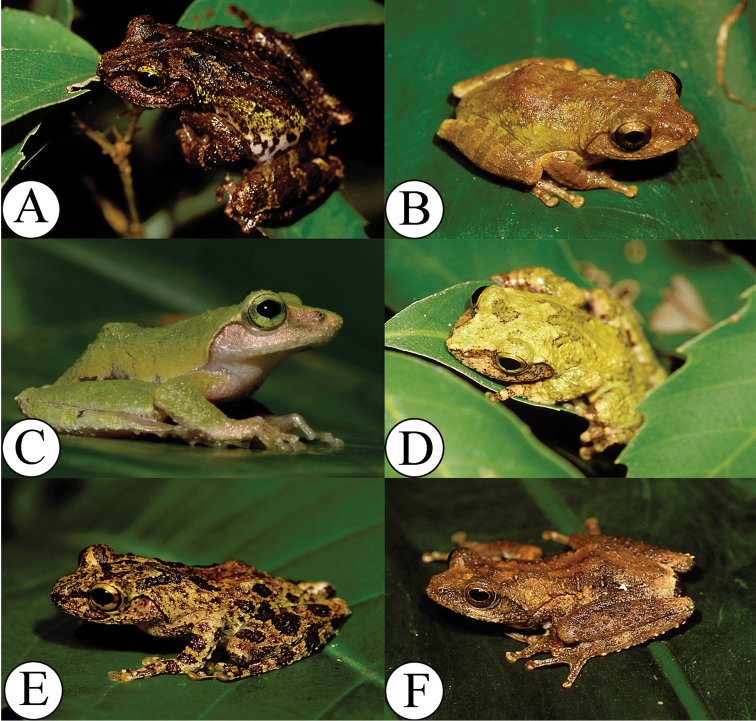
Four *Kurixalus* species of Taiwan. **A** *Kurixalus berylliniris* sp. n.(holotype, adult, dark morph) **B** *Kurixalus wangi* sp. n. (holotype) **C** *Kurixalus berylliniris* sp. n. (sub-adult) **D** *Kurixalus berylliniris* sp. n. (adult, light morph) **E** *Kurixalus eiffingeri* **F** *Kurixalus idiootocus*.

**Figure 4.**
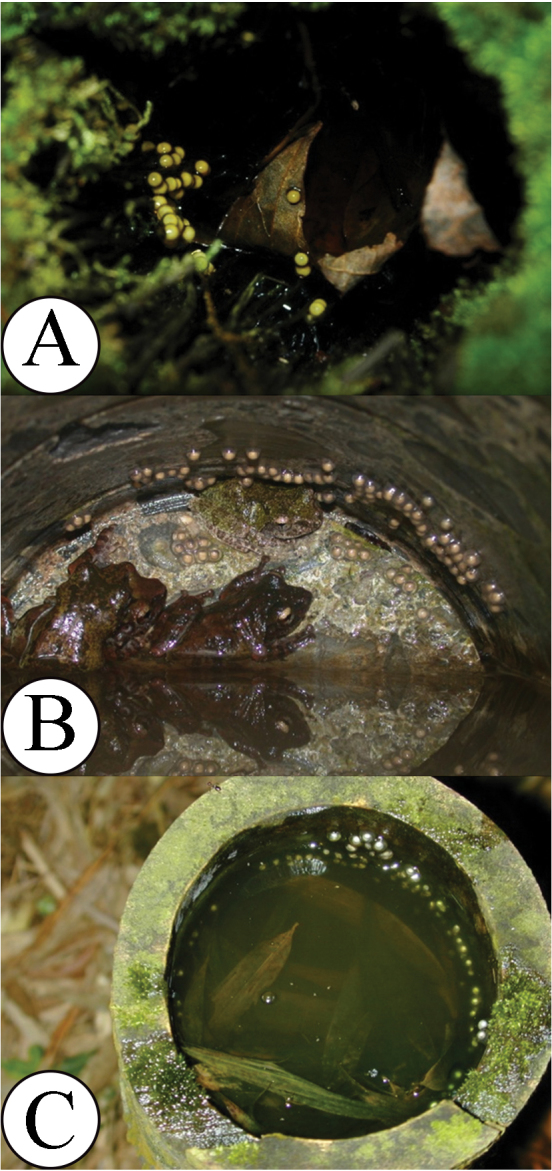
Nesting sites of three tree-hole breeding *Kurixalus* species (a nest is made by the animal). **A** eggs of *Kurixalus berylliniris* sp. n. **B** eggs of *Kurixalus wangi* sp. n.; note that the parents were present with eggs **C** eggs of *Kurixalus eiffingeri*.

**Figure 5.**
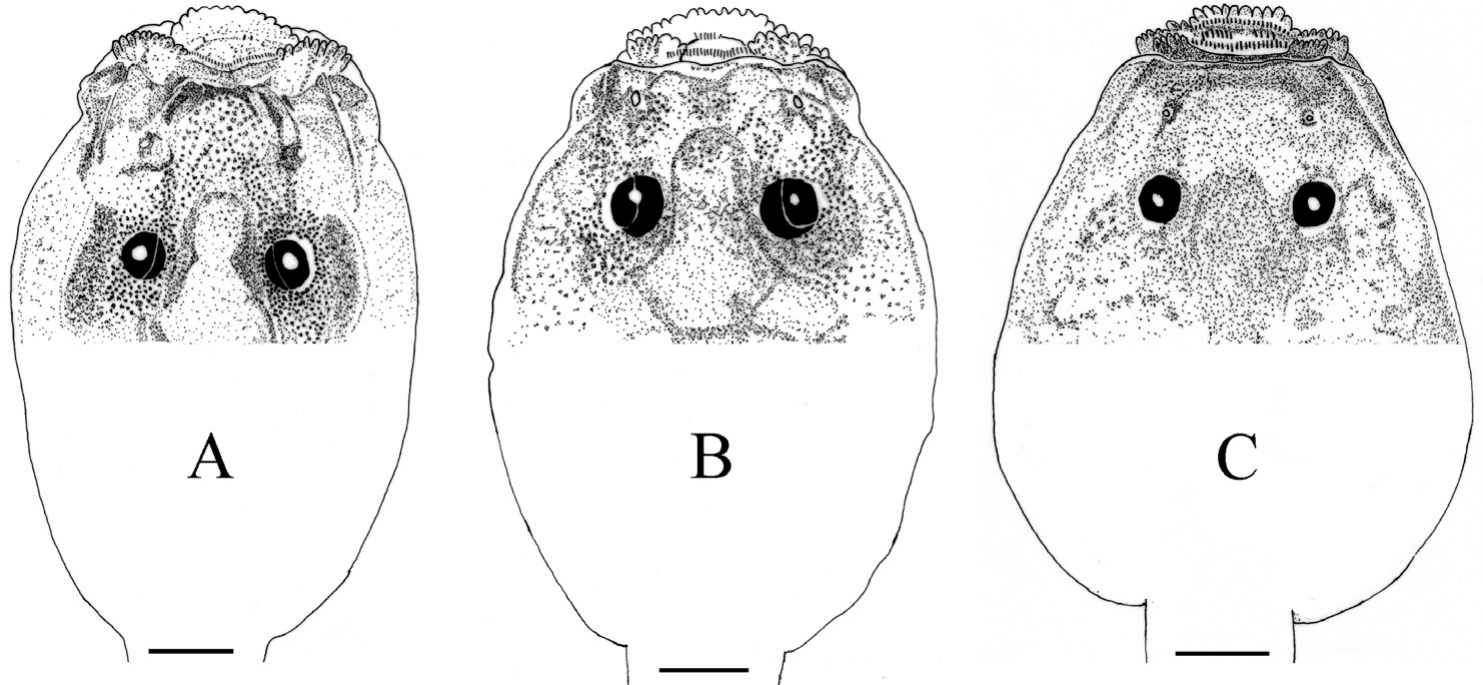
Dorsal view of tadpole head region of three oophagus *Kurixalus* species. **A** *Kurixalus berylliniris* sp. n. **B** *Kurixalus wangi* sp. n. **C** *Kurixalus eiffingeri*. Scale bars 1 mm.

**Figure 6.**
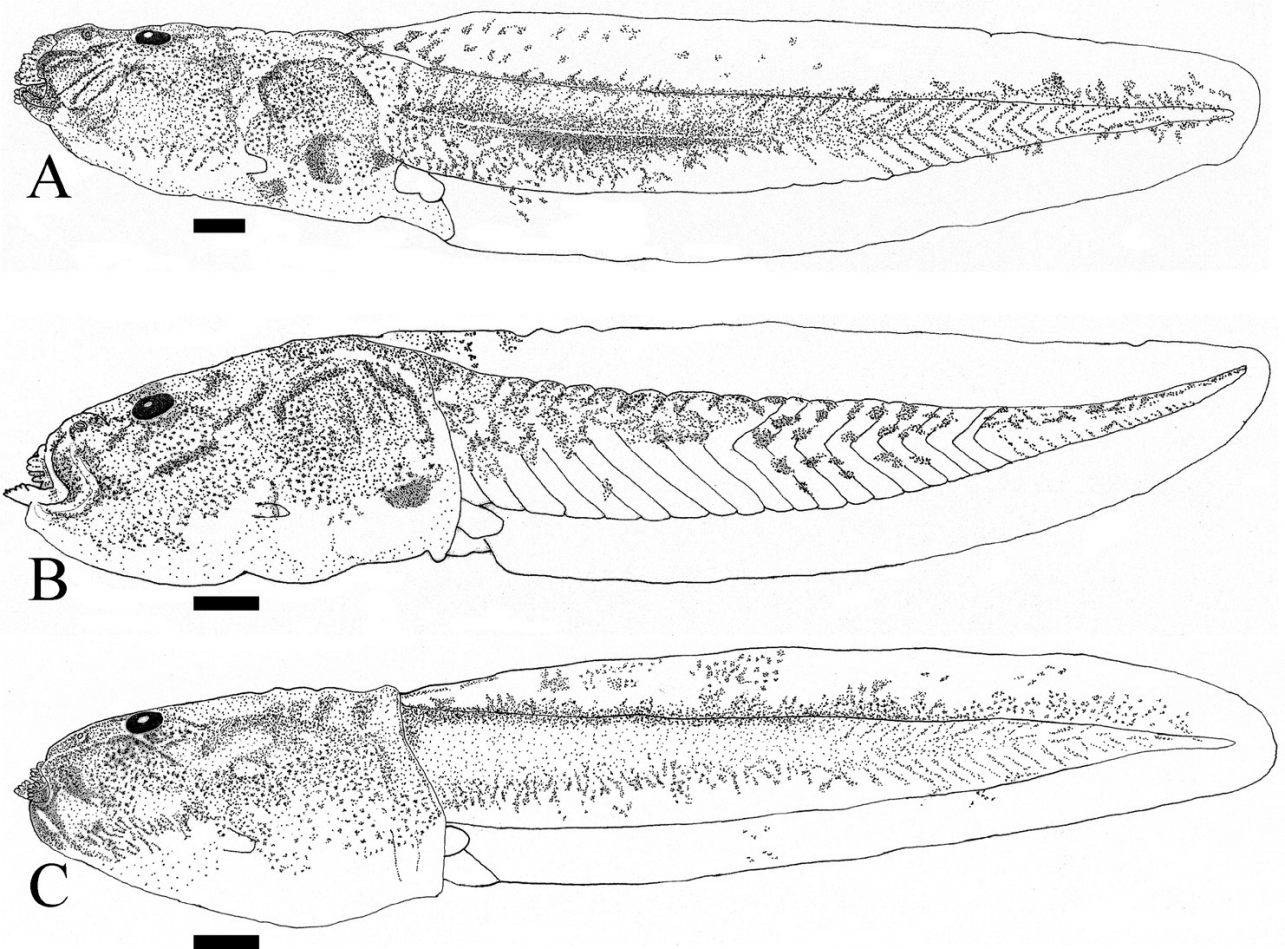
Lateral view of tadpoles of three oophagus *Kurixalus* species. **A** *Kurixalus berylliniris* sp. n. **B** *Kurixalus wangi* sp. n. **C** *Kurixalus eiffingeri*. Scale bars 1 mm.

**Figure 7.**
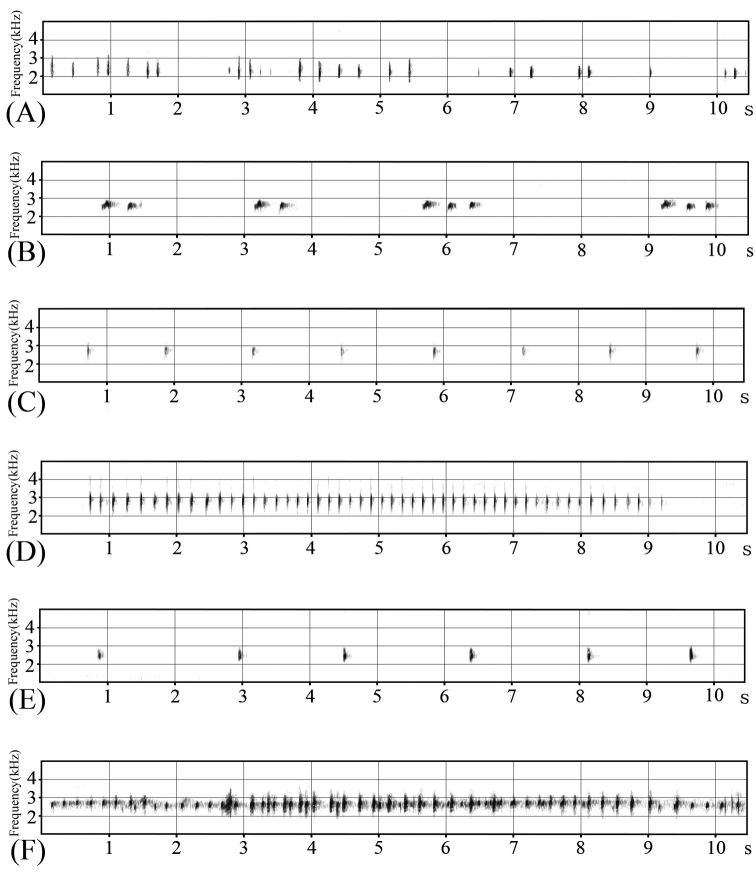
Advertisement calls of four *Kurixalus* species from Taiwan**. A**. *Kurixalus berylliniris* sp. n. “slow call” **B**. *Kurixalus berylliniris* sp. n. “rapid call” **C**. *Kurixalus wangi* sp. n. “slow call” **D** *Kurixalus wangi* sp. n. “rapid call” **E** *Kurixalus eiffingeri* **F** *Kurixalus idiootocus*.

**Figure 8.**
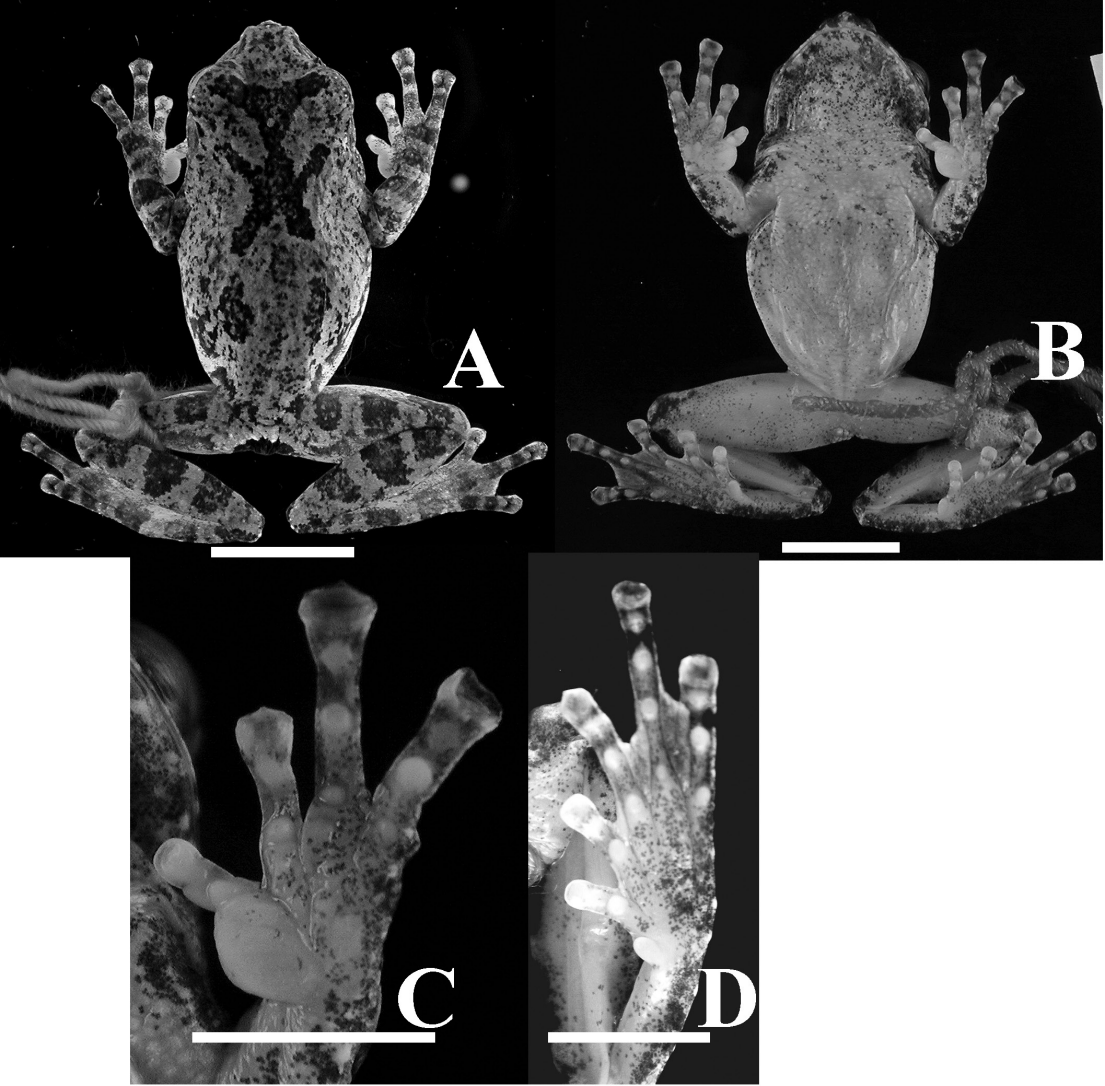
Holotype of *Kurixalus wangi* sp. n. Dorsal (**A**), ventral (**B**), and ventral view of hand (**C**) and foot (**D**). Scale bars 10 mm in (A) and (B); 5 mm in (C) and (D).

Figs 2 , 3A, C, D , 4A , 5A , 6A , 7A , 7B , 8B ; Table 1 , Table S5 , Table S6 , Table S7

##### Material examined.

**Holotype.** ASIZAM 0053, an adult male (Figs 2 and 3A, Table 1), collected on Ligia timber trail, 1250 m elevation, Taitung County, Taiwan (Fig. 1, Loc. 20, 22°49'26.79"N, 121°00'35.45"E), 15 September 2005 by Shu-Ping Wu.

**Table 1.** Measurements (in mm) of type series and other specimens of *Kurixalus berylliniris* sp. n. and *Kurixalus wangi* sp. n. Abbreviations as in Materials and methods.

	*Kurixalus berylliniris* sp. n.					*Kurixalus wangi* sp. n.				
	male	male		female		male	male		female	
no.	ASIZAM 53	Mean±SD^a^	range	Mean±SD	range	ASIZAM 55	Mean±SD^a^	range	Mean±SD	range
	holotype	(n=13)		(n=7)		holotype	(n=17)		(n=8)	
SVL	40.0	34.4±4.1	29.0–42.3	37.8±7.1	27.6–46.3	29.3	30.0±0.9	28.6–31.6	34.3±1.8	30.8–37.1
HW	13.1	11.7±1.3	7.4–13.7	12.9±1.8	10.4–14.6	11.3	11.2±0.6	10.1–12.2	12.5±0.6	11.4–13.2
HL	9.9	9.0±1.1	7.8–12.0	9.6±0.9	8.3–10.4	8.6	7.9±0.5	6.7–8.6	9.3±1.0	8.3–11.2
IN	4.5	3.6±0.5	3.0–4.5	4.0±0.7	3.1–4.7	3.5	3.3±0.2	2.9–3.7	3.7±0.3	3.2–4.2
EN	4.5	3.5±0.5	2.9–4.5	3.7±0.6	3.0–4.4	3.7	3.2±0.3	2.6–3.7	3.4±0.2	3.2–3.8
ED	5.2	4.3±0.5	3.5–5.2	4.5±0.8	3.6–5.9	3.5	4.1±0.3	3.5–4.8	4.5±0.4	4.0–5.0
UEW	2.9	3.1±0.4	2.8–4.1	3.5±0.6	2.7–4.0	3.6	3.0±0.4	2.2–3.6	3.3±0.1	3.0–3.4
DFE	7.9	6.7±0.6	6.0–7.9	7.4±1.1	5.7–8.4	6.7	6.4±0.5	5.5–7.2	6.9±0.5	6.2–7.7
DBE	11.6	10.4±1.0	8.7–12.8	11.5±1.3	9.2–13.0	11.0	9.9±0.5	9.1–11.0	11.1±0.8	9.6–12.1
IO	5.2	4.1±0.6	3.4–5.2	4.4±0.7	3.6–5.4	3.6	4.0±0.2	3.6–4.3	4.2±0.3	3.8–4.8
TAD	2.5	2.1±0.2	1.7–2.5	2.4±0.5	1.6–2.9	1.9	1.9±0.2	1.5–2.1	2.0±0.1	1.9–2.2
AXI	13.8	10.8±1.4	8.9–13.8	12.2±2.6	8.4–15.4	10.3	9.9±0.9	7.4–11.0	11.0±1.1	8.9–12.4
AGD	19.4	17.4±1.6	15.6–20.6	18.2±4.5	11.2–24.3	14.1	13.3±1.6	10.4–16.4	15.0±2.0	12.6–17.8
UAW	7.5	6.8±0.9	5.6–8.2	7.1±1.2	5.3–8.4	4.5	5.6±0.5	4.5–6.6	6.2±0.4	5.7–7.0
PAL	13.9	10.7±1.5	9.2–13.9	12.3±2.2	8.9–14.9	9.2	9.0±0.6	8.1–10.3	9.5±0.5	8.8–10.3
F1L	6.8	5.5±0.8	4.6–6.8	5.9±1.1	4.2–7.1	4.6	4.6±0.4	3.7–5.2	4.8±0.4	4.5–5.7
D3L	2.4	1.6±0.4	1.1–2.4	1.7±0.4	1.2–2.6	1.8	1.5±0.2	1.1–1.9	1.7±0.1	1.5–1.8
FEL	18.8	16.1±2.1	13.0–18.8	17.5±2.7	13.8–20.3	14.6	14.6±0.8	13.0–16.0	16.3±0.4	15.4–16.8
TBL	19.9	17.0±2.1	14.6–19.9	18.1±2.7	14.2–21.8	14.4	14.6±0.8	13.3–16.2	16.4±0.7	15.1–17.1
TSL	9.6	8.1±0.8	6.9–9.6	8.4±1.2	6.2–9.4	5.9	6.9±0.6	5.9–7.9	7.7±0.5	7.2–8.8
FOL	17.9	15.4±2.0	13.0–18.5	17.0±3.0	12.6–19.9	12.9	12.4±0.7	11.4–13.9	13.8±0.7	12.6–14.9
TL	6.7	5.7±1.0	4.5–7.2	6.3±1.5	4.3–8.0	4.2	4.3±0.2	3.8–4.6	4.8±0.4	4.3–5.3
T4D	1.7	1.2±0.3	0.8–1.7	1.3±0.4	0.8–2.0	1.1	1.3±0.2	1.0–1.6	1.4±0.3	1.0–1.7
IML	1.9	1.4±0.3	1.0–1.9	1.6±0.4	1.0–2.0	1.3	1.2±0.2	0.9–1.8	1.4±0.1	1.2–1.5
										

a including the holotype

**Paratypes.** NCHUZOOL 11311-13 collected on 2 August 2005 by Hui-Ming Huang at the type locality; NCHUZOOL 11431, ASIZAM 0054 collected on 15 September 2005 by Shu-Ping Wu at the type locality; NCHUZOOL 11442 (eggs and tadpoles), collected on 7 February 2006 by Shu-Ping Wu at the type locality; NCHUZOOL 11448, collected on 16 February 2006 by Shu-Ping Wu at 425 meters above sea level, at Antong, Hualien County (Fig. 1, Loc. 19, 23°17'06.62"N, 121°21'44.82"E).

##### Type locality.

Ligia timber trail, 1250 meters above sea level, Taitung County, Taiwan, Republic of China (Fig. 1, Loc. 20, 22°49'26.79"N, 121°00'35.45"E).

##### Diagnosis.

A moderate-sized *Kurixalus*. Females average about 41 mm snout-vent length (range: 27.6–46.3 mm); males average about 35 mm (range: 29.0–42.3 mm). Iris emerald to light green. Two dark brown spots on eyelids, separated from each other and from X-shaped blotch on dorsum. Subarticular tubercles on foot rounded and flat. Belly and throat white or faintly-speckled. Prepollex in males squarish, compressed and expanded. About half-webbed between two outer toes. Anterior margin of tadpole dorsal fin extending to body. Tadpole heavily dark brown to black pigmented in gular region and on tail muscle. Upper lip of tadpole with deep transverse furrow, and prominent ridge extending from upper lip to anterior margin of nostril (key of tadpole, 3).

##### Etymology.

The epithet *berylliniris* is a compound word formed from *beryllin* (L.), green-colored, and from *iris* (L.), iris of the eye, and is treated as a noun in nominative singular in opposition to the generic name.

##### Description of holotype.

Habitus moderately slender and somewhat flattened, size moderate (SVL 40.1 mm); head wider than long; tip of snout pointed; snout obtuse in lateral view; nostril barely visible from above; canthus rostralis curved, prominent; loreal region concave, oblique; interorbital distance 1.5 times wider than upper eyelid width; nostril oval, oblique, closer to tip of snout than to eye; internarial distance slightly longer than nostril-eye distance; eye diameter larger than nostril-eye distance; pupil horizontal; tympanic region oblique; diameter of tympanum approximately half of eye diameter; tympanum distinct, round; tympanum to eye distance smaller than half tympanum diameter; supratympanic fold from posterior tip of eye to base of arm; jaw angle almost to posterior rim of tympanum; premaxillary and maxillary teeth present; choana exposed; vomerine teeth present only on left side; tooth patch oval, about half of choana diameter. Vocal slits near commissure of jaw, slit-like.

Limbs slender; tips of all four fingers expanded into discs with ventro-marginal and transverse grooves; disc of finger III about 67% of tympanum diameter; relative finger lengths: I<II<IV<III; relative disc widths I<II<III<IV; disc on finger I small, slightly wider than phalanx width. Webbing more extensive on right hand; only trace of webbing on left hand between fingers III and IV; webbing formula on right hand: I(1.5)–(1.5)II(2)–(2)III(1)–(1.5)IV; subarticular tubercles rounded, elevated, larger under phalanges than at base of fingers; supranumerary tubercles present, smaller than subarticular tubercles; two palmar tubercles, outer longer but narrower than inner. Nuptial pad greatly expanded, proximal edge more flattened than at base; epidermal glands discontinuous, on lateral margin of nuptial pad, and on internal margin of finger I; outer margin of hand with series of longitudinal tubercles somewhat connected to weak skin folds.

Heels overlapping when adpressed; tips of toes expanded into discs with ventro-marginal and transverse grooves; relative length of toes: I<II<V<III<IV; relative width of toe discs: I<II<III<IV<V; disc on toe I small, truncated; disc widest on toe V, less than twice of width of phalanx; webbing formula: I(0.5)–(1)II(0.5)–(1.5)III(1)–(2)IV(1)–(0.5)V; subarticular tubercles rounded, elevated, those at base of toes III, IV, and V smaller than supernumerary tubercles; inner metatarsal tubercle flat, oval, median margin free; outer metatarsal tubercle absent; a series of tubercles on outer surface of tarsus to outer margin of toe V.

Dorsum granular with small tubercles; palpebral tubercles absent; flank and venter smooth or slightly shagreened.

**Color.** In preservative, two dark brown spots on eyelids; dorsum at shoulder region with a large irregular X-shaped blotch; anterior horn of blotch not continuous with spots on eyelids; two brown blotches on lower back in groin region; flank white with large irregular blotches; dark blotches at cloacal opening, surrounded ventrally by white tubercles; loreal region with dark brown irregular spot; dark spots also present under eye, on posterior part of upper lip near jaw joint, and on supratympanic fold; arm with one thick cross bar on upper arm, two on forearm, one on outer palm; three transverse bars on thigh and on tibia; medial palm and foot white on dorsal surface; venter white; few irregular brown spots on chest, faintly maculated on gular region (Figs 2 and 3A).

**Color in life.** iris emerald to light green; dorsum dark green to deep tan with a black X-shaped and irregular blotches; tympanum light yellowish-brown with small dark spots; medial surface of hand and foot creamy white; venter cream sprinkled with minute black spots in gular region (Fig. 3A, C, D).

**Variation.** Sexual dimorphism was evident in the possession of nuptial pads and the hypertrophied upper and lower arms in males. Females were 10% larger than males (t-test, *p* > 0.05). Females possess a supra-cloacal flap (absent in males). The species has dark and white morphs. The dark morph is similar to the holotype (Fig. 3A). In the white morph, the dorsum is light emerald green, and the dorsal X pattern is obscured (Fig. 3C, D). Measurements of the holotype and paratypes are shown in Table 1.

##### Description of eggs and tadpoles.

Average diameter of the eggs was 4.55 (± 0.25) mm (n = 5) with capsule and 1.79 (± 0.09) mm (n = 8) without capsule. The eggs were creamy yellow with developing embryos. The range of total length of five preserved tadpoles between stages 26–33 was 17.64–30.00 mm (Fig. 5A; Table S4).

Dorsal surface of tadpoles dark brown; ventral surface white; tail fins almost transparent with many faint black flecks; region of tail muscle heavily pigmented, especially anteriorly; body ovoid in lateral view, compressed above, more rounded below; eyes dorsal, not visible from below; eyes on anterior 1/3 of body; nostril lateral, about half way between upper lip and eye; internarial distance 105% of interorbital distance; eye-nostril distance smaller than interorbital distance; a very prominent and elevated ridge extending from nostril to upper lip; a deep transverse groove present in posterior to upper lip; a longitudinal groove on either side of head from lateral rim of upper lip to level between nostril and eye (Fig. 5A). Oral disc terminal, opening anterodorsally; lateral half of upper lip with a single row of papillae; lower lip slightly protruding; a single row of short papillae on lower lip without median interruption. Tooth row formula 3(3)/1(1) or 3(3)/0 or 3(3)/1; the first and second tooth rows on upper lip long, traverse entire width of upper labium; the third upper tooth row only visible when entire upper lip is upturned, very short, abutting lateral-most edge of second row; lower labium teeth lost in most specimens. Upper and lower beak black; upper beak straight, with median notch and moderately long lateral process, upper beak with medial transverse ridge; lower beak serrated on inner surface. Spiracle sinistral, not tubular; opening at center of body, visible in ventral aspect.

Vent dextral, opening at proximal edge of ventral fin; tail moderately strong, deeper than body; dorsal and ventral fin depth equal, almost symmetrical (or slightly deeper on dorsal fin); origin of dorsal fin anterior to that of ventral fin, on posterior 1/5 of body (Figs 5A and 6A; Table S4).

##### Distribution and ecological notes.

*Kurixalus berylliniris* sp. n. occurs in eastern Taiwan (at 225 to 1250 meters above sea level). The highest recorded elevation was on the eastern slope of the Central Mountain Range (Taitung County, 1250 meters above sea level), and the lowest recorded elevation was on the western slope of the Coastal Range (Hwalien County, 225 meters above sea level). Specimens were collected near the canopy level in moist broad-leaf forests in Taitung and on forest edges in Hwalien. The northern border of the specimen’s distribution was near the Guangfu township of the central Hualien County (Fig. 1, Green stain).

##### Mating calls.

Mating calls were heard during the winter months from November through February. Both a slow call and a rapid call consisted of a single beeping sound. Slow calls recorded in the field had an average duration of 158 (± 56) ms (n = 30, equivalent thereinafter); rapid calls had an average duration of 103 (± 42) ms. Intervals between notes were 3195 (± 1060) ms (slow calls) and 1562 (± 1442) ms (rapid calls). For the slow and rapid calls, the maximum frequencies of calls were 2901 (± 89) Hz (slow calls) and 2961 (± 71) Hz (rapid calls); the minimum frequencies of calls were 2517 (± 106) Hz (slow calls) and 2518 (± 124) Hz (rapid calls). (Fig. 7A B; Table 2).

**Table 2.** Measurements of advertisement calls of *Kurixalus* species.

Species	MAX (Hz)	MIN (Hz)	WID (Hz)	DUR (msec)	INT (msec)	DF (Hz)
***Kurixalus berylliniris* sp. n. (slow)**	2901 (89)	2517(106)	384 (80)	158 (56)	3195(1060)	2704(35)
***Kurixalus berylliniris* sp. n. (rapid)**	2961 (71)	2518 (124)	443 (97)	103 (42)	1562(1442)	2772(360)
***Kurixalus wangi* sp. n. (slow)**	3185(194)	2399 (122)	786 (192)	99 (19)	1122 (230)	2841(145)
***Kurixalus wangi* sp. n. (rapid)**	3072 (47)	2565 (62)	507 (62)	57 (15)	115 (22)	2848(59)
***Kurixalus eiffingeri***	3034 (59)	2550 (54)	484 (90)	154 (27)	2063 (121)	2772(260)
***Kurixalus idiootocus***	2889 (46)	2412 (64)	477 (80)	48 (16)	1900 (40)	2647(62)

MAX : maximum frequency ; MIN : minimum frequency ; WID : width of frequency (MAX-MIN) ; DUR : single note duration ; INT : time interval between notes ; DF : dominant frequency ; all data shown are mean and standard deviation (in parentheses) based on 30 advertisement calls recorded in the field under natural conditions. Environmental parameters: 1) *Kurixalus berylliniris* sp. n.: 18 °C, 93% RH. 2) *Kurixalus wangi* sp. n.: 25 °C, 84% RH. 3) *Kurixalus eiffingeri*: 19 °C, 91% RH. 4) *Kurixalus idiootocus*: 24 °C, 80% RH.

Eggs and tadpoles were found in the pooled water in decaying trunks of tree ferns, *Cyathea spinulosa*. The eggs were adhered together in a single layer by colloidal gel and attached to the inner roof and wall above the water. A total of 62 eggs were counted in one tree hole (Fig. 4A). Tadpoles collected at stages 31 and 33 had a creamy yellow stomach, suggesting the tadpoles might have ingested eggs recently.

#### Kurixalus wangi sp. n.

Taxon classificationAnimaliaAnuraRhacophoridae

http://zoobank.org/FED9C27A-95D9-43B5-A789-351F1F208953

Figs 3B , 4B , 5B , 6B , 7C, D , 8 ; Table 1 , Table S5 , Table S6 , Table S7

##### Material examined.

**Figure 9.**
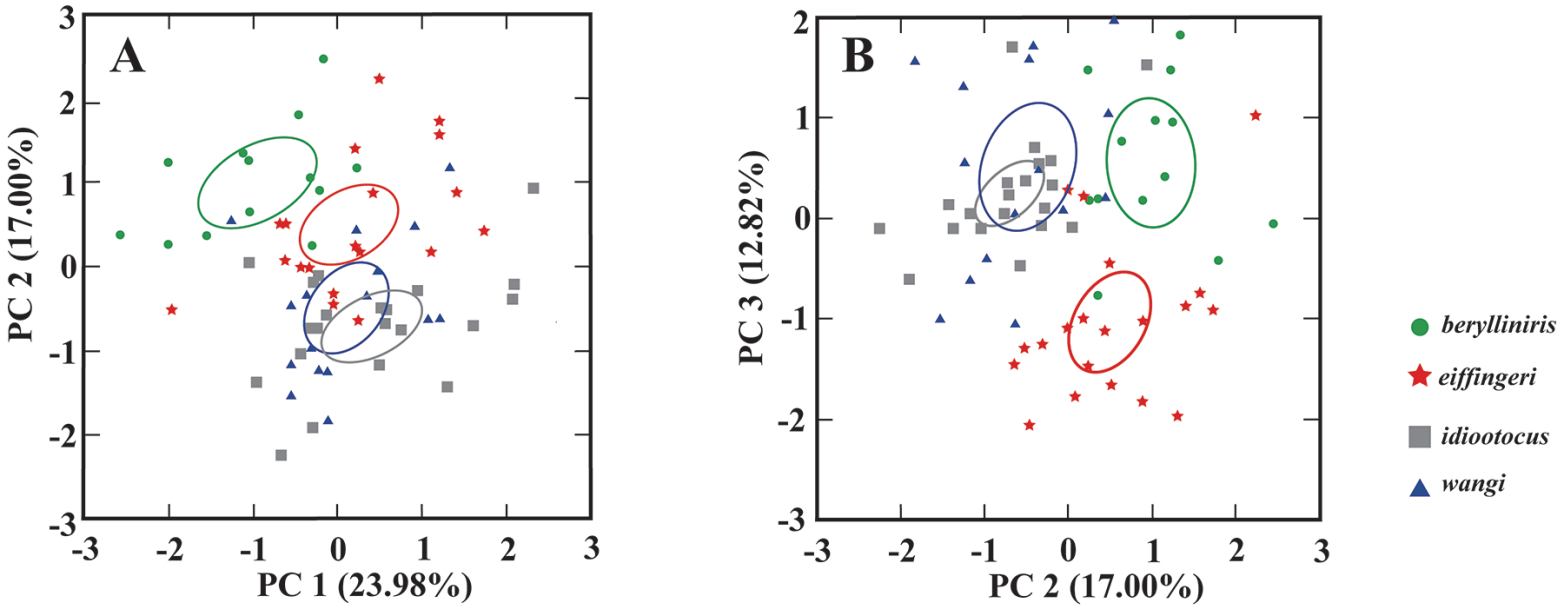
PCA morphometric comparisons of four *Kurixalus* species from Taiwan. Scatterplots of (**A**) principal components 1 and 2, and (**B**) principal components 2 and 3 of size-adjusted morphometric data for male frogs of the four *Kurixalus* species. The 95% confidence ellipses for each population (ELM) are shown.

**Holotype.** ASIZAM 0055 (Figs 3B, 9, Table 1), adult male collected on Shouka timber trail, 400 meters above sea level, Pingtong County, Taiwan (Fig. 1, Blue dots, 22°14'41.12"N, 120°49'50.14"E), 9 February 2005 by Shu-Ping Wu.

**Paratypes.** NCHUZOOL 11161–62, collected on 13 September 2005 by Sheng-Hai Wu at Shuan-Liu, Pingtung County (22°13'15.58"N, 120°49'21.92"E); NCHUZOOL 11314, 11318, 11321-32, collected on 20 October 2005 by Shu-Ping Wu, on Shouka timber trail, Pingtung County, NCHUZOOL 11315, collected on 8 December 2005 by Shu-Ping Wu at Nanjenshan, Pingtung County (22°05'08.32"N, 120°51'24.04"E); NCHUZOOL 11316-17, 11319, collected on 20 December 2005 on Shouka timber trail, Pingtung County; NCHUZOOL 11334-35, collected on 7 December 2005 by Shu-Ping Wu, on Shouka timber trail, Pingtung County; NCHUZOOL 11441 (tadpoles and eggs), ASIZAM 0056 and NCHUZOOL 11445-47, collected on 9–12 February 2006 by Shu-Ping Wu, on Shouka timber trail, Pingtung County.

##### Type locality.

Shouka timber trail, 400 meters above sea level, Pingtung County, Taiwan, Republic of China (Fig. 1, Loc. 21, 22°13'15.58"N, 120°49'21.92"E).

##### Diagnosis.

A small to moderate-sized *Kurixalus*. Females snout-vent length averaging about 34 mm (range: 30.8–37.1 mm); males averaging 30 mm (range: 28.6–31.6 mm). Iris golden-yellow. Two anterior horns of the X-shaped marking on back extending to eyelid. Webbing extensive on toes, extending to the toe disc on the inner margin of toe V. Belly and throat whitish. Anterior margin of tadpole dorsal fin extending to posterior body. Tadpole with almost no pigment on region of tail muscle. Upper lip of tadpole with shallow transverse furrow.

##### Etymology.

The epithet is named and dedicated to Mr. Ching-Shong Wang for his pioneering work and contributions to the herpetology of Taiwan (Wang 1962). Mr. Wang discovered two rhacophorid frogs (*Rhacophorus taipeianus* and *Kurixalus idiootocus*) (Liang and Wang 1963, Kuramoto and Wang 1987) in Taiwan and suggested, in the early 1980s, that some *Kurixalus* specimens collected near the type locality of this new species might be different from *Kurixalus eiffingeri* (personal communication). The name is used in the genitive case.

##### Description of holotype.

Habitus slender, body flat, small (SVL 29.3 mm), head wider than long, snout shape in dorsal view subovoid with pointed tip; profile acuminate, slightly protruding; canthus rostralis distinct, rounded; loreal region oblique, slightly concave; nostril oval and oblique; nostril closer to tip of snout than to eye; internarial distance equals nostril to eye distance; nostril to eye distance smaller than eye diameter; interorbital distance subequal to internarial distance and eyelid width; pupil horizontally oval; tympanum distinct, round, upper margin covered by curved supratympanic fold, which runs from posterior angle of eye to arm; angle of jaw at level of middle of tympanic ring; tympanum less than half of eye diameter; tympanum to eye distance greater than half tympanum diameter; premaxillary and maxillary teeth present; choana exposed; vomerine odontoid in oval patch, equal in diameter to choana; vomerine teeth present; tongue large, forked and shallowly emarginate; no lingual papilla; vocal slits long, near commissure of jaw on floor of mouth.

Limbs moderately robust; forearm shorter than hand; tips of fingers expanded into discs with ventro-marginal and transverse grooves; disc of finger III about 2/3 of tympanum diameter; finger length I<II<IV<III; disc even, truncate, with indistinct transverse groove; size of disc I<II<III<IV; disc of finger I small, same width as phalanx width; phalanges emarginate with trace of webbing; subarticular tubercles prominent, rounded, globular; prepollex expanded, rounded; glandular skin associated with nuptial pad from base of disc I on medial and dorsal side of pollex; palmar tubercle double, oval, subequal in size; supernumerary tubercles small; outer margin of fourth finger with longitudinal flat tubercles connected into a weak flap.

Heels overlapping when adpressed; hind limb moderate in length; shank shorter than thigh and longer than foot; tips of toes expanded into discs with ventro-marginal and transverse grooves; relative length of toes I<II<III<V<IV; relative size of discs I<II<III<IV<V; disc on toe I same width as phalanx width; discs truncate and with indistinct transverse grooves. Webbing moderate on all toes; webbing formula I(1)–(trace)II(0.5)–(1.5)III(1)–(2)IV(1)–(0.2)V; weak dermal fringe on outer side of toe V, from posterior tarsus to base of disc V, formed by continuous elongated papilla; subarticular tubercles rounded, slightly conical; subarticular tubercle on proximal joint on toe IV smaller than the others; inner metatarsal tubercle oval, small; outer metatarsal tubercle absent; supernumerary tubercle absent; small white-tipped tubercle on heel.

Skin shagreen, tubercles not present on back; ventral surface slightly granular, white tipped dermal tubercles on posterior thigh. Series of tubercles near lateral margin of upper eyelids; skin smooth on flank; white tipped tubercles on lateral lower arm in ventral view.

**Color.** In preservative, dorsum grayish with black irregular spots; patches of dark brown markings on median eyelid, forming triangular X-shaped blotch; two posterior branches of the X marking terminated at middle of dorsum; two dark blotches on posterior back; flank with dark oblique irregular band demarcating grayish dorsum and whitish venter; dark irregular blotches on loreal region, antero-ventral corner of eye, and tympanum; black band from anterior eye angle, through nostril to tip of snout; gular region sprinkled with black spots; upper arm with three wide bands, thigh and shank with three bands; ventral surface orange, speckled with brown spots on gular region; vent with large dark brown blotch over cloacal opening, surrounded ventrally and dorsally by white tubercles (Figs 3B and 8).

Color in life, iris golden-yellow; dorsum dark brownish-green scattered with deep brown and black spots, with dark X marking on anterior half of dorsal surface; tympanic membrane light brown to milk-white; white and rounded tubercle located on outer fringe of heel (Fig. 3B).

**Variation.** Females were 14% larger than males (Table 1) (t-test, *p* < 0.01). Males had longer hands than females. Sexual dimorphism is evident in the possession of nuptial pads and the hypertrophied upper and lower arms in males. Females possess supracloacal dermal flaps (absent in males). The dorsum color of both genders ranges from light brown with distinctly dark markings to almost uniformly light green. Webbing patterns between the two outer metatarsals vary. Among the 69 specimens examined, one was not webbed, and two were 2/3 webbed, and the rest of the specimens were half-webbed or less. Measurements of holotype and other type specimens are shown in Table 1.

##### Description of eggs and tadpoles.

Average diameter of eggs from 4 clutches was 3.37 (± 0.27) mm with capsule (n = 38) and 1.74 (± 0.09) mm without capsules; eggs were creamy yellow with developing embryos. The range of total length of ten tadpoles between stages 27–32 was 13.19–22.64 mm (Fig. 4B; Table S4).

Dorsal surface of tadpoles dark brown; ventral surface white; pigment on tail confined mostly to upper half of tail muscle; tail fins transparent; body ovoid in lateral view, flat and sloping above, rounded below; eye dorsal, not visible from below, located on anterior 1/3 of body; nostril lateral; distance from nostril to upper lip much shorter than to eye; internarial distance subequal to interorbital distance; eye-nostril distance less than internarial distance. Face with slightly elevated ridge, from rostrum to upper lip (Fig. 5B). Oral disc terminal, opening anteriorly; lower lip slightly protruding; lateral half of upper lip with a single row of papilla; a single row of short papillae on lower lip without median interruption. Tooth row formula 3(3)/2(1); the first and second rows on upper lip long, traverse entire width of upper labium; the third upper tooth row only visible when the entire upper lip is upturned, very short, confined to the lateral-most edge of the upper labium. The first tooth row on lower labium interrupted medially by a gap half the width of the lower jaw, the second tooth row short, less than half of oral disc width. Lower beak visible only in youngest tadpoles, black in color, upper beak straight, with very short lateral processes, upper beak ridged in middle; lower beak serrated on inner surface; older tadpoles all have broken upper beaks, lower beak white; spiracle sinistral, not tubular; opens at center of body, visible in ventral aspect. Vent dextral, opening at lower edge of ventral fin; tail deeper than body at center; dorsal and ventral fin equal in depth, tail muscle moderately strong. Dorsal fin origin on posterior body (Figs 5B and 6B).

##### Distribution and ecological notes.

*Kurixalus wangi* sp. n. is distributed in the southern part of Pingtung County in southern Taiwan below 500 meters above sea level (Fig. 1, Blue dots). All specimens were collected in the shrubs of secondary forests or lowland broad-leaved forests at low altitudes.

##### Mating calls.

Mating calls were heard in bushes or on tree branches up to 3 m above the ground between September and March, peaking in December. A slow call and a rapid call were identified. Both types of call consisted of a single beeping sound. Slow calls recorded in the field had an average duration of 99 (± 19) ms (n = 30, equivalent hereafter) and rapid calls had an average duration of 57 (± 15) ms. Intervals between notes were 1122 (± 230) ms (slow calls) and 115 (± 22) ms (rapid calls). For the slow and rapid calls, the maximum frequencies of calls were 3185 (± 194) Hz (slow calls) and 3072 (± 47) Hz (rapid calls); the minimum frequencies of calls were 2399 (±122) Hz (slow calls) and 2565 (± 62) Hz (rapid calls). (Fig. 7C, D; Table 2). The diagrams of all *Kurixalus* species’ calls from Taiwan are illustrated in Fig. 7 and detailed in Table 2.

Eggs were discovered in tree hollows, plastic pipes embedded in retaining walls on slopes (Fig. 4B), and discarded plastic cups on the forest floor. Eggs of some clutches adhered in a single layer to the walls above water; others were submerged in water. The average clutch size was 70 (n = 7, range: 56–104). Tadpole stomachs contained yellow yolky substances, suggesting that the tadpoles might have ingested eggs recently.

##### Morphological comparisons.

Within-species comparisons showed that the body size was differentiated by sex in *Kurixalus eiffingeri* and *Kurixalus wangi* sp. n., but not in *Kurixalus berylliniris* sp. n. (Table 1, Table S5). Among-species comparisons indicated that males were significantly different in body size (p < 0.001, Table S5). ANCOVA tests showed that the three species were significantly different in all morphometric characteristics. For the ANCOVA of male morphometric data, all characters differed significantly, and 17 characteristics exhibited significant variation in size-adjusted means (Table S6). In females, all characteristics also differed in slopes, and eight characteristics exhibited significant variation in size-adjusted means. A repeated ANOVA comparing the two new species and *Kurixalus eiffingeri* showed that all morphometric characteristics differed significantly among species between males and females (Table S6).

In the PCA, after eliminating the effect of size by using a normalizing ratio (measurements divided by SVL) and omitting the five non-normal morphometric characteristics (HL, EN, TAD, D3L, TL), 23.98% of the variation was associated with body size (Table S7). The large-sized *Kurixalus berylliniris* sp. n. was separated from the other three species, while *Kurixalus idiootocus* and *Kurixalus wangi* sp. n. overlapped considerably (Fig. 9A). In the plot of two shape components (Fig. 9B, Table S7), *Kurixalus berylliniris* sp. n. and *Kurixalus eiffingeri* are clearly differentiated from the other two species *Kurixalus idiootocus* and *Kurixalus wangi* sp. n.

##### Mating call comparisons.

The calls of the two new species and of *Kurixalus eiffingeri* were found to be different in maximum frequency, single note duration, and time interval between notes of mating calls. The minimum frequency among the three species was not different (Table 3).

**Table 3.** Comparisons of the characteristics of slow mating calls among *Kurixalus eiffingeri*, *Kurixalus berylliniris* sp. n. and *Kurixalus wangi* sp. n.

Characteristics	*Kurixalus eiffingeri* vs. Kurixalus berylliniris*	Kurixalus berylliniris* vs. Kurixalus wangi*	*Kurixalus eiffingeri* vs. Kurixalus wangi*
	WMWODDS (95% CI)	WMWODDS (95% CI)	WMWODDS (95% CI)
Maximum frequency	3.4 (1.59–10)	Inf (Inf-Inf)	2.67 (1.391–5.88)
Minimum frequency	1.2 (0.571–2.67)	0.18 (0–0.54)	0.53 (0.25–1.04)
Single note duration	0.63 (0.29–1.32)	0 (0–0)	0.37 (0.17–0.72)
Time interval between notes of mating	0.07 (0–0.22)	0 (0–0)	0 (0–0)
Width of frequency	13.67 (4.5-Inf)	Inf (Inf-Inf)	12.75 (5.11-Inf)
Dominant frequency	2.38 (1.2–6.33)	3 (1–9)	1.5 (0.77–3.23)

* slow call

### Phylogenetic relationships

**Figure 10.**
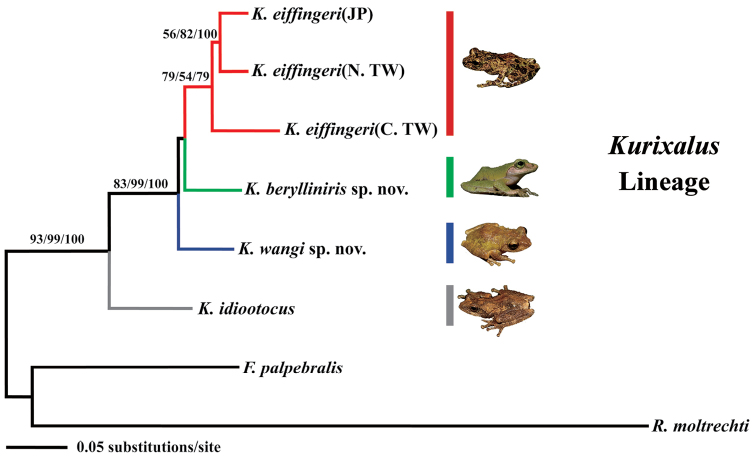
Phylogenetic relationship of all *Kurixalus* species from Taiwan. A phylogram showing the phylogenetic relationships of the four *Kurixalus* species, obtained by a maximum likelihood search based on 1207 nucleotides from mtDNA CO1 and 16S rRNA genes. *Feihyla palpebralis* and *Rhacophorus moltrechti* were used as outgroups. The three values on each branch are maximum likelihood (ML), maximum parsimony (MP), and neighbor-joining (NJ) analyses with bootstrapping support based on 2000 replicates. Bootstrapping values below 50% are not shown. (JP: Ryukyu Islands of Japan; N. TW: northern Taiwan; C. TW: central Taiwan).

**Figure 11.**
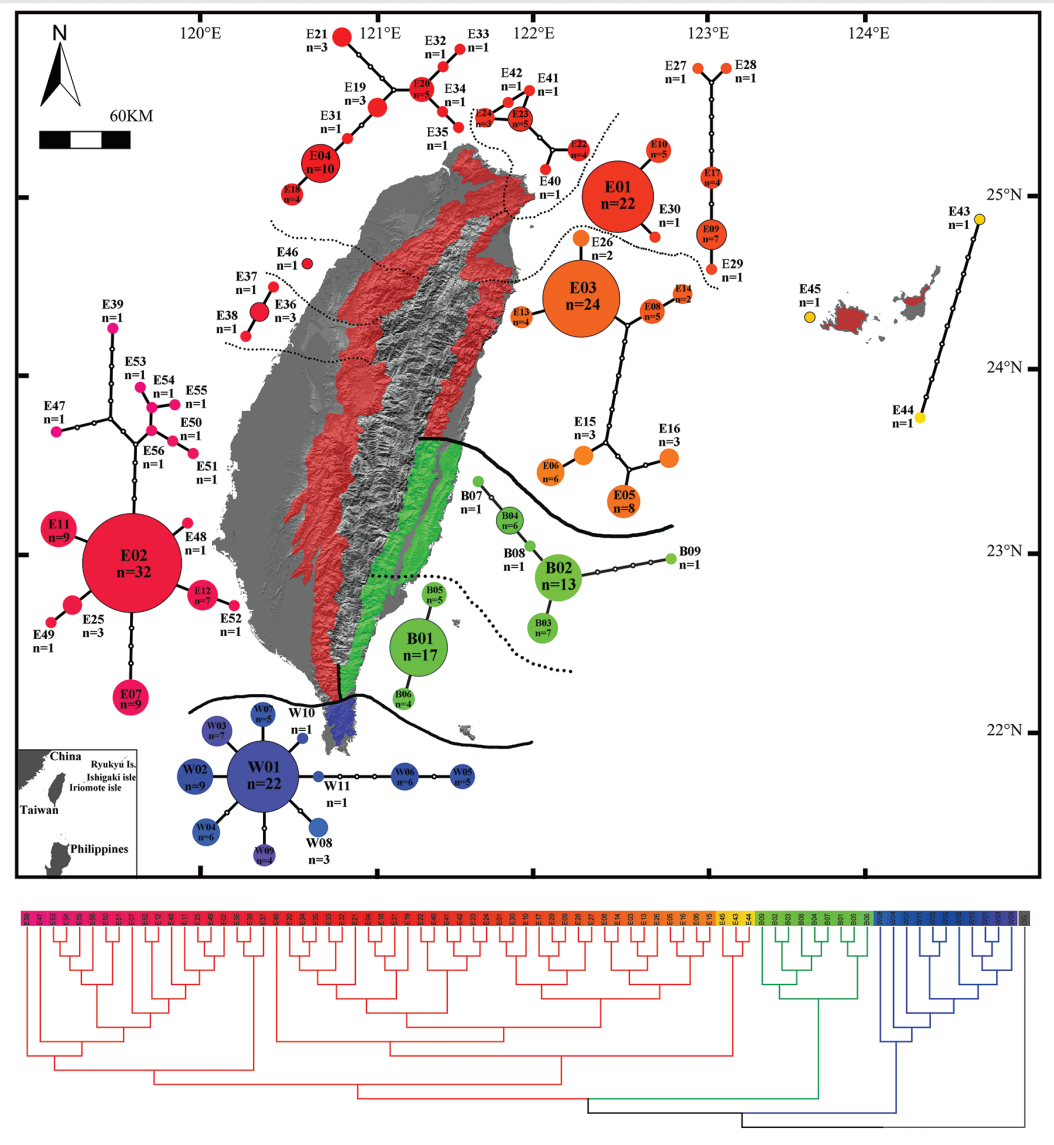
Geographic distribution and genetic structures of *Kurixalus eiffingeri* and the two newly discovered cryptic species from Taiwan and its two adjacent islands. Red: *Kurixalus eiffingeri*; Green: *Kurixalus berylliniris* sp. n.; B: *Kurixalus wangi* sp. n. Bold lines mark the boundaries of each species’ distribution, dotted lines discriminate different genetic groups intra species. Below: a consensus ML tree to show the variation between haplotypes. GenBank number accession numbers KT259055–KT259131.

As demonstrated by the high bootstrap support, the robustness of the phylogenetic relationship of the three rhacophorid genera is strong. Based on this robust phylogenetic tree, we found that the among-genera genetic distances were greater than the within-genus genetic distance (Fig. 10). Using the partial sequence of mtDNA CO1 gene as a molecular marker (Table S2), the genetic distances of the all pair-wise comparisons of the four *Kurixalus* species were all larger than 10% (Table S3). The phylogenetic trees constructed by Bayesian inference, NJ analysis, and MP methods showed the same topology (Fig. 10). The topology of branches was sufficiently supported by the posterior probabilities, bootstrap values, and branch lengths. The four *Kurixalus* species of Taiwan formed a well-structured monophyletic group with distinguishable branch length. Samples of *Kurixalus eiffingeri* collected from Iriomote Island, northern Taiwan, and central Taiwan were embedded in the same lineage and formed a monophyletic group (Fig. 11 below). Individuals from southern (*Kurixalus wangi* sp. n.) and eastern Taiwan (*Kurixalus berylliniris* sp. n.) were sister taxa of *Kurixalus eiffingeri*. *Kurixalus idiootocus* was phylogenetically distinct from the three *Kurixalus* species (Figs 10 and 11 below).

The phylogenetic consensus tree of *Kurixalus wangi* sp. n. presented a star-like haplotype minimum spanning network with a core ancestral haplotype (W01) and ten derivative haplotypes (W02-W11) (Fig. 11, blue haplotypes, above; blue clade, below; GenBank accession numbers KT259064–KT259074). The within-species haplotypes of *Kurixalus berylliniris* sp. n. and *Kurixalus eiffingeri* showed a transitional variation pattern. *Kurixalus berylliniris* presented two genetic groups that showed two genetic subgroups (Fig. 11, green haplotypes, above; green clade, below; GenBank accession numbers KT259055–KT259063). *Kurixalus eiffingeri* (including the populations in Taiwan and Ryukyu islands) revealed several subgroups based on genetic structures (Fig. 11, red haplotypes, above; red clade, below; GenBank accession numbers KT259075–KT259130). The topology of the phylogenetic tree matched the geographic distributions of the populations. The monophyletic lineage coincided with the phylogenetic tree, and the genetic divisions are shown in Figs 10, 11 and Table S3.

The indicators of genetic diversity – haplotype diversity (Hd), nucleotide diversity (Pi), and number of haplotypes of each population – are shown in Table S8 and Fig. 11. Pairwise comparisons of *Fst* and *Nm* in three *Kurixalus* species (Table S9) demonstrated extremely low gene flow among the three species.

### Key to the species of Kurixalus from Taiwan

**Table:** 

1	Dorsum with a brown “) (“ saddle-shaped marking, two arms of the marking not touching each other at mid-dorsum; venter with two large brownish rounded blotches in axilla region; males with very weak nuptial pad; iris golden speckled with brown; cloacal opening of female without supracloacal flap	***Kurixalus idiootocus***
–	Dorsum with a X- or Y-shaped marking, two arms of the marking touching each other at mid-dorsum; venter without blotches; males with greatly expanded nuptial pad; cloacal opening in females with supracloacal flap	**2**
2	Belly smooth; two spots present on upper eyelids, separated from each other, not in contact with marking on back; medial palmar tubercle larger than lateral one; iris emerald to light green	***Kurixalus berylliniris* sp. n.**
–	Belly granular or shagreened; spots on upper eyelids in contact each other, forming a dark bar or connecting with the X-marking on back; two palmar tubercles equal in width; iris golden	**3**
3	Tubercles on lateral margin of finger IV connected with dermal fringe; venter whitish with very little pigmentation; loreal region oblique; canthus rostralis curved	***Kurixalus wangi* sp. n.**
–	Tubercles on lateral margin of finger IV separated from each other; venter with numerous fine brownish dots, especially in the gular region; loreal region vertical; canthus rostralis straight	***Kurixalus eiffingeri***

### Key to the tadpoles of the genus Kurixalus species from Taiwan

**Table:** 

1	Lentic tadpole, mouth antero-ventral, tooth formula 5(3-5)/3 or 5(2-5)/3	***Kurixalus idiootocus***
–	Oophagous tadpole, mouth terminal or antero-dorsal, tooth row three or less on upper lip, two or less in lower lip	**2**
2	Dorsal fin originates at base of tail muscle	***Kurixalus eiffingeri***
–	Dorsal fin originates on posterior body	**3**
3	Dorsum flat in profile; nostril equidistant between upper lip and eye; deep transverse groove on upper lip; a ridge present from lateral margin of upper lip to nostril; gular region and tail muscle heavily pigmented	***Kurixalus berylliniris* sp. n.**
–	Dorsum sloping in profile; nostril closer to upper lip than to eye; inconspicuous transverse groove on upper lip; no ridge from lateral margin of upper lip to nostril; gular region and tail muscle without pigmentation, or with only small scattered spots	***Kurixalus wangi* sp. n.**

## Discussion

Based on the 1) different mating call characteristics, 2) different timing of mating calls, 3) diversified morphological characteristics and genetic composition, 4) no interspecies gene flow indicated by extremely high *Fst* and low *Nm*, and 5) sufficient genetic divergences among species (Vences et al. 2005a, b, Table S3), we concluded that the two *Kurixalus* taxa (*Kurixalus berylliniris* sp. n. and *Kurixalus wangi* sp. n.) from eastern and southern Taiwan are two distinct species. In contrast, with our re-evaluation of the taxonomic status of *Kurixalus effingeri* we confirmed that the *Kurixalus effingeri* populations in northwestern Taiwan, central Taiwan, and the Iriomote and Ishigaki isles (Ryukyu islands) are a robust genetic monophyletic group (Figs 10 and 11, Red clade). The two new species resemble *Kurixalus eiffingeri* in breeding habits, tadpole morphology, and clutch size. Therefore, *Kurixalus berylliniris* sp. n. and *Kurixalus wangi* sp. n. were cryptic members of the *Kurixalus eiffingeri* complex before our study.

Unlike previous researchers who did not note the within-species variation of mating calls (Kuramoto 1974, Kuramoto and Wang 1987), using an advanced voice recording system we identified that the mating calls of these three species were different in maximum frequency, width of frequency, single note duration, and time interval between notes of the mating calls. The divergence of mating calls plays a major role in pre-zygotic isolation—an important component of speciation (Mayr and Ashlock 1991). Speciation is further promoted by the two new species having different reproductive seasons (Mayr 1942, Mayr and Ashlock 1991, Coyne and Orr 2004)

The guts of tadpoles of the two new species contained a yellow ‘yolky’ substance. When the same characteristic was observed in *Kurixalus eiffingeri* it was confirmed as tadpole oophagy (Ueda 1986, Liang et al. 2002). Therefore, it is likely that the tadpole oophagy is a synapomorphy for the two new species and *Kurixalus eiffingeri*. Interestingly, *Kurixalus idiootocus*, as well as all known *Kurixalus* species from mainland China and Southern Asia lack this particular reproductive behavior. Therefore, the oophagy reproductive behavior could also support the phylogenetic positions of *Kurixalus eiffingeri*, *Kurixalus berylliniris* sp. n., and *Kurixalus wangi* sp. n. within the *Kurixalus* genus.

Previous reports estimated the distribution of *Kurixalus eiffingeri* to be up to 2000 m in mountain forests all over the island of Taiwan. These records were problematic in that they primarily relied on mating call surveys. Our study not only demonstrated the usefulness of advanced voice recording systems in identifying the new species but also highlighted the importance of collecting voucher specimens. In addition to the two newly described species and *Kurixalus eiffingeri*, there is one further species in this genus, *Kurixalus idiootocus*. Until 1987, *Kurixalus idiootocus* was treated as a subgroup within *Kurixalus eiffingeri* (Kuramoto and Wang 1987, Liang and Wang 1963). This species is found in low hill habitats up to 1000 meters above sea level throughout the island of Taiwan except the eastern part. In our study, we assessed the morphological characteristics and genetic structure of the four species within the genus *Kurixalus* and confirmed the four Taiwanese *Kurixalus* species are phylogenetic monophyly. To our knowledge, this is the first comprehensive report of the genus *Kurixalus* on the island of Taiwan. The actual amphibian species diversity on the island of Taiwan is likely higher than currently thought, given the diverse habitats and the dynamic history of geographic events. Although Taiwan is a highly developed island with significant alterations to the natural landscape and destruction of critical habitats for amphibians, it is noteworthy that during the last fifty years, six of the seven newly described frog species in Taiwan were treefrogs inhabiting forested areas.

## Author contributions

SP Wu envisioned the original idea, executed this study and wrote the manuscript; CC Huang helped with the statistical methods, data analysis, and paper writing; CL Tsai and TE Lin performed the data analyses; JJ Jhang measured and analyzed the mating call data; SH Wu described adult and tadpole morphology, performed anatomical studies, morphometric analyses and proofread the paper. All the authors contributed to this paper sufficiently.

## Supplementary Material

XML Treatment for Kurixalus berylliniris

XML Treatment for Kurixalus wangi

## Appendix

### Additional specimens examined

***Buergeria japonica***: NCHUZOOL: 4021, 4825, 4826 (Taiwan). ***Buergeria robusta***: NCHUZOOL 4025, 4027, 4831 (Taiwan). ***Kurixalus eiffingeri***: NTU 927–28, 931-32, 939, 1052, 1054–56, 1649 (Shitou, Nantou); 1058-67, 1645–46, 1769 (Wulai, Taipei); NCHUZOOL: 2502–03, 2508, 2509(C/S), 2514–15, 2517–19, 2523–24, 2525(C/S), 2526–27, 2535, 2536(C/S), 2537, 2538(C/S), 2539–40, 2545–47, 2550–51, 2829, 2831, 4018(C/S), 11320, 11436–40 (Shindian, Taipei); 11333 (Shitou, Nantou). ***Kurixalus idiootocus***: NTU 929, 1005–09, 1033, 1035–37, 1038–42 (Shiding, Taipei); 1045 (Suao, Yilan); 1010 (holotype of *Chirixalus idiootocus*), 1011, 1013–29, 1657, 1695–96, (Yanminshan, Taipei); 1658 (Shindien, Taipei); 1708–09 (Juchi, Chiayi); 1770 (Wulai, Taipei); NCHUZOOL 1010, 2780, 2782, 2784, 2785(C/S), 2787–88, 2792, 2795, 2796(C/S), 2805, 2845, 2847, 2954, 2956(C/S), 2959–63, 3709, 3711(C/S), 4304(C/S), 4990, 4993, 4995(C/S), 7402. ***Kurixalus wangi* sp. n.**: NTU 1043–1044, 1046, 1647-48 (Manjo, Pingtung County).

### Supporting information

**Table S1.** Morphometric characteristics and the abbreviations in this study.

Abbreviation	Morphometric characteristic
SVL	snout-vent length
HW	head width
HL	head length
IN	internarial distance
EN	eye-narial distance
ED	horizontal eye diameter
DFE	distance between the anterior margins of eyes
DBE	distance between the posterior margins of eyes
UEW	upper eyelid width
IO	interorbital distance
TAD	tympanic annulus diameter
AXI	between posterior margins of the upper arm
AGD	axilla-groin distance
UAW	forearm length
PAL	manus length
F1L	ength of first finger from base of palmar tubercle to tip of third finger disc
D3L	width of third finger disc
FEL	femur length
TBL	tibia length
TSL	tarsus length
FOL	foot length from proximal margin of inner metatarsal tubercle to tip of fourth toe
TL	first toe length
IML	inner metatarsal tubercle length
T4D	disc width of fourth toe

**Table S2.** DNA sequences used in this study and their GenBank accession numbers.

Species	Sampled locality	Gene sequences	No. of GenBank	Voucher specimen
*Kurixalus eiffingeri*	Ryukyu Islands, Japan	mtDNA CO 1	DQ468681	NTUMA 2427
		mtDNA 16S	DQ468673	
*Kurixalus eiffingeri*	Wulai, Taipei, northen Taiwan	mtDNA CO 1	DQ468680	NCHUZOOL 11320
		mtDNA 16S	DQ468672	
*Kurixalus eiffingeri*	Xitou, Nantou, central Taiwan	mtDNA CO 1	DQ468678	NCHUZOOL 11333
		mtDNA 16S	DQ468670	
*Kurixalus berylliniris* sp. n.	Beinan, Taitung, eastern Taiwan	mtDNA CO 1	DQ468677	ASIZAM 00053
		mtDNA 16S	DQ468669	
*Kurixalus wangi* sp. n.	Shouka, Pintung, southern Taiwan	mtDNA CO 1	DQ468679	ASIZAM 00055
		mtDNA 16S	DQ468671	
*Kurixalus idiootocus*	Wulai, Taipei, northern Taiwan	mtDNA CO 1	DQ468682	NA
		mtDNA 16S	DQ468674	
*Feihyla palpebralis*	Yunnan, China	mtDNA CO 1	DQ468683	NA
		mtDNA 16S	DQ468675	
*Rhacophorus moltrechti*	Antong, Hualien, eastern Taiwan	mtDNA CO 1	DQ468684	NA
		mtDNA 16S	DQ468676	

**Table S3.** Genetic distances among *Kurixalus* species and two outgroup taxa in CO 1 gene (above diagonal) and 16S rRNA gene (below diagonal). Numbers on top row refer to species shown on the left column. Genetic distances are shown as percentage.

Species	1	2	3	4	5	6	7	8
1. *Kurixalus eiffingeri* (JP)	–	6.30	9.13	11.23	10.85	15.02	21.36	24.99
2. *Kurixalus eiffingeri* (NTW)	0.92	–	9.48	9.92	10.47	16.21	20.51	25.94
3. *Kurixalus eiffingeri* (CTW)	3.41	3.21	–	10.84	13.12	14.20	20.51	24.33
4. *Kurixalus berylliniris* sp. n.	3.19	3.00	4.77	–	10.65	14.36	19.21	23.89
5. *Kurixalus wangi* sp. n.	3.80	3.60	4.19	3.98	–	14.16	21.17	25.06
6. *Kurixalus idiootocus*	5.97	5.97	5.76	6.15	6.59	–	18.18	23.06
7. *Feihyla palpebralis*	10.68	10.90	11.56	11.34	11.13	11.61	–	23.79
8. *Rhacophorus moltrechti*	14.04	13.12	14.74	14.48	14.49	14.94	15.40	–

Abbreviations: *K*: *Kurixalus*; *F*: *Feihyla*; *R*: *Rhacophorus*; JP : Ryukyu Islands of Japan ; NTW : northern Taiwan ; CTW : central Taiwan .

**Table S4.** Comparisons of measurements and ratios of tadpoles of three oophagus *Kurixalus* species from Taiwan (ANOVA significant level, *: 0.01 < p < 0.05; **: p < 0.01).

species	*berylliniris*	*wangi*	*eiffingeri*	ANOVA
measurements	n=5	n=10	n=8	
TL	24.89 ± 5.21	18.60 ± 2.44	16.75 ± 2.85	**
BL	7.92 ± 1.72	6.68 ± 1.01	5.54 ± 1.01	**
CL	16.97 ± 3.51	11.92 ± 1.79	11.22 ± 1.87	*
TH	4.84 ± 1.10	4.04 ± 0.84	2.94 ± 0.66	**
TM	1.99 ± 0.39	1.86 ± 0.38	1.63 ± 0.31	
NA	1.99 ± 0.21	1.59 ± 0.23	1.30 ± 0.20	**
IN	1.89 ± 0.51	1.59 ± 0.27	1.38 ± 0.29	*
MW	1.94 ± 0.71	1.57 ± 0.38	1.37 ± 0.28	
BL / TL	0.32 ± 0.01	0.36 ± 0.04	0.33 ± 0.01	*
CL / TL	0.68 ± 0.01	0.64 ± 0.04	0.67 ± 0.01	*
TH / TL	0.19 ± 0.01	0.22 ± 0.04	0.17 ± 0.01	*
TM / TL	0.08 ± 0.00	0.10 ± 0.02	0.10 ± 0.00	*
NA / TL	0.08 ± 0.01	0.09 ± 0.04	0.08 ± 0.00	
IN / TL	0.08 ± 0.01	0.09 ± 0.01	0.08 ± 0.00	
MW / TL	0.08 ± 0.01	0.08 ± 0.01	0.08 ± 0.01	
TH / CL	0.28 ± 0.02	0.34 ± 0.08	0.26 ± 0.02	*
TM / MW	1.08 ± 0.21	1.20 ± 0.11	1.19 ± 0.11	
IN / NA	0.94 ± 0.16	0.99 ± 0.05	1.06 ± 0.08	
CL / BL	2.15 ± 0.10	1.81 ± 0.27	2.03 ± 0.11	**
TH / TM	2.42 ± 0.19	2.18 ± 0.18	1.80 ± 0.09	**

**Table S5.** Body size variation (SVL) in female and male *Kurixalus* species from Taiwan (mean ± standard deviation). (**: significant level < 0.01).

Species	female	male	t-test
*Kurixalus berylliniris* sp. n.	37.8±7.1	34.4±4.1	0.178
	n = 7	n = 13	
*Kurixalus eiffingeri*	33.7±2.9	31.1±2.3	**
	n = 15	n = 20	
*Kurixalus wangi* sp. n.	34.3±1.8	30.0±0.9	**
	n = 8	n = 17	
*Kurixalus idiootocus*	36.3±2.5	26.9±1.4	**
	n = 3	n = 21	
ANOVA	F_3,29_ = 1.964	F_3,67_ = 30.227**	

**Table S6.** Analysis of covariance of morphometric characteristics of males and females among four *Kurixalus* species.

measurement	*berylliniris* sp. n.	*eiffingeri*	*idiootocus*	*wangi* sp. n.	equal slope	equal mean
male						
HW	10.59	10.83	10.56	11.21	**	0.001
HL	8.16	8.04	8.21	7.96	**	
IN	3.29	3.41	3.20	3.31	**	0.028
EN	3.08	2.91	2.98	3.21	**	0.004
ED	3.95	4.10	4.27	4.11	**	
UEW	2.82	3.12	3.27	3.03	**	0.019
DFE	6.28	6.23	6.25	6.38	**	
DBE	9.55	9.76	9.67	9.99	**	0.046
IO	3.69	3.81	3.95	4.00	**	0.005
TAD	1.93	1.33	1.76	1.87	**	
AXI	9.25	9.72	9.82	9.93	**	
AGD	15.47	14.48	14.91	13.42	**	0.000
UAW	6.12	5.40	5.70	5.60	**	0.000
PAL	9.56	10.10	9.21	9.06	**	0.000
F1L	4.95	5.06	4.34	4.60	**	0.000
D3L	1.30	1.86	1.78	1.56	**	0.000
FEL	14.50	14.00	13.74	14.64	**	0.010
TBL	15.51	14.59	13.99	14.62	**	0.002
TSL	7.37	6.94	6.78	6.93	**	0.028
FOL	13.76	13.66	12.94	12.48	**	0.000
TL	5.00	5.00	4.43	4.29	**	0.000
T4D	0.99	1.51	1.32	1.33	**	0.000
IML	1.27	1.36	1.39	1.23	**	
female						
HW	12.20	12.22	12.16	12.67	**	0.030
HL	9.17	9.08	8.88	9.37	**	
IN	3.77	3.75	3.73	3.74	**	
EN	3.53	3.31	3.25	3.48	**	
ED	4.25	4.56	4.05	4.52	**	
UEW	3.28	3.46	3.48	3.33	**	
DFE	7.02	7.02	6.93	7.02	**	
DBE	10.99	10.97	10.61	11.17	**	
IO	4.19	4.27	4.57	4.30	**	
TAD	2.20	2.23	2.21	2.09	**	
AXI	11.27	11.43	11.57	11.22	**	
AGD	16.49	16.94	17.55	15.36	**	
UAW	6.65	6.36	6.89	6.31	**	
PAL	11.47	11.35	10.22	9.71	**	0.000
F1L	5.52	5.52	5.08	4.92	**	0.002
D3L	1.62	2.04	1.94	1.72	**	0.005
FEL	16.51	15.60	15.93	16.53	**	0.025
TBL	17.21	16.66	16.27	16.56	**	
TSL	8.01	7.74	8.06	7.83	**	
FOL	15.83	15.35	14.93	14.08	**	0.000
TL	5.85	5.79	5.25	4.94	**	0.001
T4D	1.17	1.65	1.81	1.46	**	0.001
IML	1.48	1.49	1.60	1.43	**	

Means adjusted to SVL. * significant difference at 0.95 level, ** significant differences at the 0.99 level, exact probabilities are given only for slopes that are unequal. Sample sizes listed in Table S5.

**Table S7.** Factor loadings of the first three principal components of 18 size-adjusted morphometric characteristics of males of four *Kurixalus* species. Absolute values of loadings greater than 0.50 in boldface.

Character	PC 1	PC 2	PC 3
Eigenvalue	4.316	3.061	2.308
% variation	23.98	17	12.82
HW	**0.609**	-0.299	0.081
IN	**0.615**	0.147	-0.237
ED	**0.590**	-**0.512**	0.041
UEW	**0.581**	-0.471	-0.144
DFE	**0.742**	-0.292	0.198
DBE	**0.739**	-0.424	0.054
UEW	0.449	-0.273	0.245
AXI	0.107	0.014	-0.354
AGD	-0.156	0.162	-0.123
UAW	0.112	0.089	**0.682**
PAL	0.418	**0.604**	-**0.520**
F1L	0.293	**0.698**	-0.323
FEL	0.431	0.413	0.479
TBL	0.453	**0.584**	**0.527**
TSL	0.300	**0.507**	**0.502**
FOL	0.413	**0.686**	-0.150
T4D	**0.533**	-0.018	-**0.564**
IML	**0.582**	0.015	-0.206

**Table S8.** Genetic variation of three *Kurixalus* species from Taiwan according to mtDNA *CO1* gene partial sequence.

	N	Number of haplotypes	Haplotype diversity (Hd)	Nucleotide diversity (Pi)
*Kurixalus berylliniris* sp. n.	55	9	0.82088	0.02271
*Kurixalus wangi* sp. n.	69	11	0.85209	0.00443
*Kurixalus eiffingeri*	219	53	0.94600	0.05524

**Table S9.** *Fst* (above diagonal) and *Nm* (below diagonal) between three *Kurixalus* species from Taiwan.

	*Kurixalus berylliniris* sp. n.	*Kurixalus wangi* sp. n.	*Kurixalus eiffingeri*
*Kurixalus berylliniris* sp. n.	--	0.85922	0.60226
*Kurixalus wangi* sp. n.	0.04	--	0.71956
*Kurixalus eiffingeri*	0.17	0.10	--

*Fst* = 1 / (4*Nm*+1) whereas *Nm* =((1/*Fst*)-1)/4.
